# Postoperative C-Reactive Protein as a Marker of the Early Inflammatory Response in Robotic Versus Laparoscopic Minimally Invasive Surgery for Endometrial Cancer: A Propensity Score-Matched Analysis

**DOI:** 10.3390/jcm15197376

**Published:** 2026-09-23

**Authors:** Masahiro Kagabu, Takayuki Nagasawa, Rina Shibata, Shunsuke Tatsuki, Nanako Jonai, Shun Takagi, Sho Sato, Eriko Takatori, Yoshitaka Kaido, Tadahiro Shoji, Tsukasa Baba

**Affiliations:** Department of Obstetrics and Gynecology, Iwate Medical University School of Medicine, 2-1-1 Idaidori, Yahaba, Iwate 028-3695, Japan; tnagasaw@iwate-med.ac.jp (T.N.); rina96rina11@gmail.com (R.S.); 412.sailing@gmail.com (S.T.); nn.jonai617@gmail.com (N.J.); came0921@gmail.com (S.T.); satos@iwate-med.ac.jp (S.S.); takatori@iwate-med.ac.jp (E.T.); ykaido@iwate-med.ac.jp (Y.K.); tshoji@iwate-med.ac.jp (T.S.); babatsu@iwate-med.ac.jp (T.B.)

**Keywords:** robotic surgery, laparoscopic surgery, endometrial cancer, C-reactive protein, early inflammatory response, propensity score matching, mediation analysis

## Abstract

**Background/Objectives:** Robot-assisted laparoscopic surgery (RALS) for endometrial cancer achieves outcomes comparable to those of conventional laparoscopy (Lap) but often requires a longer operative time, which may increase the early postoperative inflammatory response. We compared the postoperative day 1 (POD1) C-reactive protein (CRP) elevation between RALS and Lap and examined whether it was associated with the surgical platform or with patient- and procedure-related factors. **Methods:** This retrospective single-center study included 301 patients who underwent minimally invasive hysterectomy for endometrial cancer (Lap, *n* = 202; RALS, *n* = 99; 2018–2025). The primary outcome was CRP rise (POD1 minus the preoperative CRP level). Propensity score matching using preoperative covariates only (1:2, caliper 0.15 SD), multivariable regression, a formal mediation analysis with operative time as the mediator, and a test of the interaction between body mass index (BMI) category and surgical approach were performed. **Results:** RALS was associated with a greater CRP rise (2.88 vs. 2.13 mg/dL; *p* = 0.001) and a longer operative time (271.0 vs. 210.5 min; *p* < 0.001). In the matched cohort the median CRP rise was 2.91 mg/dL after RALS and 2.22 mg/dL after Lap, and the association persisted in a weighted model that accounted for the matched sets (β = 0.131; *p* = 0.023). On multivariable analysis, operative time (β = 0.0026 per minute; *p* < 0.001) and BMI (β = 0.0090 per kg/m^2^; *p* = 0.033) were associated with the CRP rise, whereas the surgical approach was not (β = −0.029; *p* = 0.623). Mediation analysis showed an indirect effect through operative time of +0.192 (95% CI, 0.120 to 0.278). The interaction between BMI category and surgical approach was not statistically significant (*p* = 0.750). **Conclusions:** The greater POD1 CRP rise after RALS was attenuated after accounting for operative time, and the mediation analysis was consistent with an indirect pathway through operative duration. This pathway cannot be established causally, because operative time could not be separated from surgeon experience, the learning curve and the era of each platform. A difference of this magnitude has no established clinical correlate, and no BMI-specific effect was demonstrated. Whether improving operative efficiency reduces the early postoperative inflammatory response should be evaluated prospectively.

## 1. Introduction

Endometrial cancer is one of the most common gynecological malignancies in developed countries, and surgical resection is the primary treatment. Japanese clinical practice guidelines have established a framework for the standard surgical management of the disease. In recent years, minimally invasive surgery has become increasingly important as it reduces perioperative complications and facilitates postoperative recovery [1]. A particular challenge in this patient population is the high prevalence of obesity, which is associated with multiple perioperative difficulties, including anesthetic management, exposure of the surgical field, positioning of the patient, and wound-related complications [2]. Against this background, both laparoscopic and robot-assisted surgeries have been established as important minimally invasive approaches for endometrial cancer. However, in obese patients or in those with significant comorbidities, the specific clinical advantages and limitations of each approach require individual evaluation [3,4].

To date, comparative studies of robot-assisted and laparoscopic surgery for endometrial cancer have primarily examined perioperative outcomes such as estimated blood loss, conversion rate to laparotomy, length of hospital stay, complication rates, and operative time. Randomized controlled trials and systematic reviews generally demonstrate that robot-assisted surgery has safety profiles comparable to those of laparoscopic surgery and suggest potential technical advantages in certain clinical situations [5,6]. Restricted analyses of obese patients have indicated that robot-assisted surgery may reduce the need for conversion to laparotomy. However, the relative merits of these two approaches have been inconsistent across studies. Important confounding factors included the operative time and patient selection [3,7]. Regarding postoperative inflammatory responses, studies have reported differences in the trajectory of inflammatory markers such as C-reactive protein (CRP) and interleukin-6 (IL-6) following surgery for endometrial cancer between surgical approaches. Nevertheless, studies directly comparing perioperative changes in CRP levels from the preoperative to early postoperative periods in robot-assisted versus laparoscopic approaches in real-world clinical settings are limited [8,9,10]. Furthermore, recent investigations into gynecologic laparoscopic and robotic surgery have shown that postoperative CRP levels are influenced by the magnitude of the surgical insult, body mass index (BMI), and operative time. This underscores the need to disentangle the independent effects of the surgical approach from the contributions of patient- and procedure-related factors [11]. Beyond its value as a marker of tissue injury, perioperative inflammatory response may be relevant to cancer biology. Surgical trauma activates damage-associated molecular pathways and induces the cytokine release, particularly IL-6, which subsequently stimulates hepatic CRP production. Experimental studies have suggested that an excessive wound-healing response may transiently impair antitumor immunosurveillance, recruit inflammatory monocytes, promote neutrophil extracellular trap formation, and facilitate the survival or outgrowth of residual tumor cells [12,13]. Clinical studies on gastrointestinal malignancies have also associated high or sustained postoperative CRP levels with recurrence. However, such an association has not been established for endometrial cancer [14].

In light of these considerations, the present study aimed to compare early postoperative inflammatory responses in patients who underwent minimally invasive hysterectomy for endometrial cancer using a robot-assisted or laparoscopic approach. The primary outcome measure was the elevation of CRP levels from the preoperative value to postoperative day 1 (POD1). In addition to examining the differences in the surgical approach between the groups, we investigated how patient- and procedure-related factors, including BMI and operative time, influence CRP elevation. Our objective was to identify independent determinants of early postoperative inflammatory responses.

## 2. Patients and Methods

### 2.1. Study Design and Patients

This retrospective single-center cohort study included all consecutive patients who underwent minimally invasive hysterectomy with bilateral salpingo-oophorectomy for endometrial cancer at Iwate Medical University Hospital between December 2018 and February 2025. Cases with a final histological diagnosis other than endometrial cancer were excluded: cervical cancer (*n* = 2), leiomyosarcoma (*n* = 1), and two patients in whom carcinoma was never demonstrated (atypical endometrial hyperplasia on both the diagnostic biopsy and the hysterectomy specimen in one, and atypical endometrial hyperplasia on biopsy with no residual lesion in the specimen in the other). Patients in whom carcinoma had been demonstrated on the diagnostic biopsy but no residual carcinoma was found in the hysterectomy specimen (*n* = 7), and four patients who underwent surgery after neoadjuvant chemotherapy, were retained; sensitivity analyses for both decisions are presented in Supplementary Table S3. The final analytic cohort comprised 301 patients: 202 in the laparoscopic group (Lap) and 99 in the robotic group (robot-assisted laparoscopic surgery [RALS]; da Vinci Si, *n* = 52; da Vinci Xi, *n* = 43; hinotori, *n* = 4). The number of missing observations for every variable, together with the distribution of cases by calendar year and robotic platform, is reported in Supplementary Table S1; no imputation was performed, analyses were carried out on complete cases for the variables concerned, and the sample size is stated for every analysis. This study was approved by the Ethics Review Committee of Iwate Medical University (approval number: MH2024-150; approval date: 25 August 2025).

### 2.2. Surgical Technique

Laparoscopic hysterectomy was performed using a standard four-trocar transperitoneal technique with the patient in a steep Trendelenburg position. RALS was performed using the da Vinci Si (until February 2023) or da Vinci Xi system (from March 2023; both Intuitive Surgical, Sunnyvale, CA, USA) with identical oncological principles. Pelvic lymph node dissection and/or sentinel lymph node biopsy were performed according to preoperative risk stratification. Four cases performed using the hinotori robotic system (Medicaroid, Kobe, Japan) were included in the overall RALS group for comparative analyses. Operative time was defined for both approaches as the interval from skin incision to the completion of skin closure; for robotic procedures this interval includes docking and console time, and this is stated explicitly because it is directly relevant to the interpretation of the study. All RALS procedures were performed or supervised by a single attending surgeon, whereas laparoscopic procedures were performed by 13 surgeons in the same department.

### 2.3. CRP Measurement and Primary Endpoint

Serum CRP levels were measured as part of routine perioperative care preoperatively (within 7 days before surgery) and on POD1. The primary endpoint, CRP rise, was defined as: CRP rise (mg/dL) = POD1 CRP − preoperative CRP. All CRP measurements were performed in the central clinical laboratory of our hospital using an immunonephelometric assay. The same assay method and measurement platform were used throughout the entire 2018–2025 study period, with no change in methodology; measurements are therefore directly comparable across the study period. Cases with a negative CRP rise (elevated preoperative CRP due to pre-existing inflammation) were retained as valid values.

### 2.4. Outcomes

The primary outcome was the CRP rise, defined as the POD1 CRP level minus the preoperative CRP level. The secondary outcomes were operative time, estimated blood loss, POD1 CRP, length of hospital stay, and readmission. No other clinical outcomes, including infectious complications, were systematically recorded in this cohort, and none are reported here.

### 2.5. Propensity Score Matching (PSM)

Propensity scores were estimated using multivariable logistic regression with the surgical approach (RALS = 1, Lap = 0) as the dependent variable and preoperative covariates only: age (continuous), body mass index (BMI, continuous), preoperative CRP (continuous), the preoperative risk category, and lymphadenectomy, which is planned before surgery on the basis of that risk category. The preoperative risk category was adapted from the recurrence-risk classification of the 2023 Japan Society of Gynecologic Oncology guidelines for the treatment of uterine body neoplasm [1]. Because the guideline classification is assigned from the final surgical pathology, we applied the same framework before surgery, substituting the histological diagnosis of the endometrial biopsy for the final histology and the preoperative stage assessed by imaging for the pathological stage: low risk, endometrioid carcinoma G1 or G2 with preoperative stage IA; intermediate risk, endometrioid carcinoma G3 or a special histological type with preoperative stage IA, or endometrioid carcinoma G1 or G2 with preoperative stage IB; and high risk, all other combinations. Atypical endometrial hyperplasia was grouped with endometrioid G1/G2 for this purpose. This preoperative adaptation, rather than the guideline classification itself, is the categorization used at our institution to plan the extent of surgery, and it is therefore available at the time the surgical approach is chosen. The postoperative FIGO stage, which was used in the original analysis, was not included because it is not available at the time the surgical approach is chosen and is a post-treatment variable. Four patients whose preoperative biopsy was not evaluable could not enter the model, leaving 297 patients eligible for matching. Matching was performed using a 1:2 nearest-neighbor algorithm without replacement with a caliper of 0.15 × standard deviation of the logit-transformed propensity score. Covariate balance was assessed by standardized mean differences (SMDs) before and after matching rather than by *p* values, with an absolute SMD below 0.10 taken to indicate acceptable balance, and is displayed as a Love plot in Supplementary Figure S1. The outcome analysis in the matched cohort accounted for the 1:2 matched-set structure using a weighted linear model with cluster-robust variance estimation by matched set and, as a check, a matched-set fixed-effects model. Full details of the propensity score model are given in Supplementary Table S2.

### 2.6. Statistical Analysis

Continuous variables were reported as medians (IQRs) and compared using the Mann–Whitney U test. Normality was assessed formally by the Shapiro–Wilk test and by inspection of histograms and Q–Q plots; the CRP variables, operative time and estimated blood loss all departed significantly from normality, and non-parametric methods were therefore used consistently for all continuous variables so that the presentation would be uniform across the tables. Categorical variables were reported as counts (percentages) and compared using the chi-square test or Fisher’s exact test, as appropriate. For multivariable analysis, CRP rise was transformed as log (CRP rise + 1) to reduce right skewness and analyzed using multiple linear regression. One patient whose preoperative CRP was markedly elevated (20.35 mg/dL) had a negative CRP rise (−16.69 mg/dL), for which the logarithmic transformation was undefined; this patient was excluded from the regression models only, leaving 300 patients. Because excluding a patient on purely computational grounds is undesirable, two sensitivity analyses that retain all 301 patients were performed using the inverse hyperbolic sine transformation and a rank-based normal score, neither of which requires positive values, together with an analysis of POD1 CRP adjusted for the preoperative CRP, which avoids the difference variable altogether (Supplementary Table S3). The primary model included the surgical approach, age, BMI, preoperative CRP, operative time, and lymphadenectomy as explanatory variables; continuous covariates were mean-centered. Because operative time lies on the causal pathway between the surgical approach and the inflammatory response, a model containing operative time estimates a direct effect conditional on operative time rather than the total effect of the surgical approach; a model omitting operative time is therefore also reported. To quantify this pathway explicitly, a mediation analysis was performed with the surgical approach as the exposure, operative time as the mediator, and log (CRP rise + 1) as the outcome, adjusted for age, BMI and lymphadenectomy, with bias-corrected and accelerated bootstrap confidence intervals from 2000 resamples. Regression coefficients (β), standard errors, and *p* values were reported, and 95% confidence intervals were derived as β ± 1.96 × standard error. Subgroup analyses were stratified by BMI category according to the World Health Organization classification (<25.0, 25.0–29.9, and ≥30.0 kg/m^2^), and the interaction between BMI category and the surgical approach was tested formally. All tests were two-sided, and statistical significance was set at *p* < 0.05. All statistical analyses were performed using EZR (version 1.68; Saitama Medical Center, Jichi Medical University, Saitama, Japan), which is a graphical user interface for R (version 4.3.3; R Foundation for Statistical Computing, Vienna, Austria); propensity score matching, cluster-robust variance estimation and the bootstrap mediation analysis used the R packages MatchIt (version 4.5.5), sandwich (version 3.1.0), lmtest (version 0.9-40) and boot (version 1.3-30). EZR is a modified R Commander that integrates statistical functions widely applied in biostatistics [15].

## 3. Results

### 3.1. Patients Characteristics

The baseline characteristics of the unmatched cohort are shown in Table 1. The RALS group was significantly younger (median 54.0 vs. 57.0 years; *p* = 0.027) and had a higher BMI (27.19 vs. 25.00 kg/m^2^; *p* = 0.032), whereas the preoperative CRP did not differ between the groups (*p* = 0.084). The distribution of FIGO stage did not differ significantly (*p* = 0.472); Stage IA disease was present in 81.8% of the RALS group and 71.8% of the Lap group. Neither the preoperative (*p* = 0.424) nor the postoperative (*p* = 0.485) histological distribution differed between the groups. The preoperative risk category, assigned from the biopsy histology and the preoperative stage, differed between the groups (*p* = 0.027): low-risk disease accounted for 91.8% of the RALS group and 79.5% of the Lap group, and intermediate-risk disease for 6.2% and 16.0% respectively. Lymphovascular space invasion was observed in 12.9% of Lap and 7.1% of RALS patients (*p* = 0.190). Lymphadenectomy was performed in 122 of 202 laparoscopic procedures (60.4%) and in 54 of 99 robotic procedures (54.5%), a difference that was not statistically significant (*p* = 0.399); the median number of nodes retrieved was 11 in both groups (*p* = 0.812). A blank entry in our database denotes that lymphadenectomy was not performed rather than an unrecorded count, so this variable is complete for all 301 patients. That the two groups underwent lymphadenectomy at a comparable rate suggests that this measured component of surgical complexity was broadly similar between the groups, although residual differences in overall case complexity cannot be excluded.

The distribution of recurrence risk categories differed significantly between the groups (*p* = 0.044). Low-risk disease accounted for 75.8% of the RALS group and 61.3% of the Lap group, whereas intermediate-risk disease was more frequent in the Lap group (26.1% vs. 17.2%). The RALS group therefore comprised a lower-risk population than the Lap group. The recurrence-risk category is assigned from the final surgical pathology and is therefore a postoperative variable; it was retained as a descriptive baseline characteristic and was not included in the propensity score model. Both approaches were performed in every year of the study, although the proportion of robotic cases increased over time, and the robotic platform changed from the da Vinci Si to the da Vinci Xi during 2023 (Supplementary Table S1).

### 3.2. Perioperative Outcomes (Unmatched)

Table 2 presents the perioperative outcomes in the unmatched cohort. RALS was associated with a significantly greater CRP rise, the primary outcome (2.88 vs. 2.13 mg/dL; *p* = 0.001). Among the secondary outcomes, operative time was significantly longer in the RALS group (271.0 vs. 210.5 min; *p* < 0.001), POD1 CRP was higher (3.14 vs. 2.40 mg/dL; *p* = 0.001), and the length of hospital stay differed statistically although the medians were identical in both groups (both 5 days; *p* = 0.014); this difference reflects the distribution within a narrow range and is of no clinical consequence. Estimated blood loss did not differ significantly between the groups (23.0 vs. 20.0 mL; *p* = 0.324). Readmission occurred in 3 of 99 RALS patients (3.0%) and in none of the 202 Lap patients (*p* = 0.035); the three events were postoperative bleeding in a patient receiving corticosteroids for polymyalgia rheumatica, a retroperitoneal haematoma with abscess formation on postoperative day 11 in a patient with poorly controlled diabetes, and pyelonephritis two weeks after surgery in a further patient with diabetes.

### 3.3. PSM

After propensity score matching, 92 patients in the RALS group were matched with 154 patients in the Lap group (Table 3). Balance is presented as standardized mean differences rather than *p* values: every covariate in the model had an absolute standardized mean difference below 0.10 after matching, the largest being 0.063 for the intermediate preoperative risk category (Table 3 and Supplementary Figure S1). Descriptively, the median CRP rise in the matched cohort was 2.91 mg/dL after RALS and 2.22 mg/dL after Lap (*p* = 0.010), and operative time remained longer (284.0 vs. 211.5 min; *p* < 0.001); these comparisons do not account for the matched-set structure. In the matched-set analysis, a weighted linear model of log (CRP rise + 1) with cluster-robust variance by matched set gave β = +0.131 (standard error 0.057; *p* = 0.023), and a matched-set fixed-effects model gave β = +0.131 (*p* = 0.016). The direction and magnitude of the primary result were therefore unchanged by the revised matching procedure.

### 3.4. Multivariable Analysis of CRP Rise

Table 4 presents the multivariable linear regression analysis of the log-transformed CRP rise (*n* = 300). Operative time was the variable most strongly associated with the outcome (β = 0.0026 per minute; 95% CI, 0.0018 to 0.0035; *p* < 0.001). BMI was also independently associated with a greater CRP rise (β = 0.0090 per kg/m^2^; 95% CI, 0.0008 to 0.0173; *p* = 0.033). In contrast, after adjustment for operative time, the surgical approach was not independently associated with the CRP rise (β = −0.0293; 95% CI, −0.1463 to 0.0876; *p* = 0.623). Age (*p* = 0.761), preoperative CRP (*p* = 0.546) and lymphadenectomy (*p* = 0.152) were not significantly associated with the CRP rise. Because operative time lies on the pathway between the surgical approach and the inflammatory response, this model estimates a direct effect conditional on operative time; in the corresponding model that omits operative time, which estimates the total effect, the surgical approach was associated with a greater CRP rise (β = 0.1591; 95% CI, 0.0538 to 0.2643; *p* = 0.003). The attenuation of the coefficient when operative time is added is consistent with an indirect pathway through operative duration but does not by itself demonstrate one.

To quantify this pathway formally, a mediation analysis was performed with the surgical approach as the exposure, operative time as the mediator, and log (CRP rise + 1) as the outcome, adjusted for age, BMI and lymphadenectomy. The robotic approach was associated with an operative time that was 72.6 min longer, and each additional minute of operative time was associated with an increase of 0.0026 in log (CRP rise + 1). The indirect effect through operative time was +0.192 (bias-corrected bootstrap 95% CI, 0.120 to 0.278), which excludes zero, whereas the direct effect was −0.032 (95% CI, −0.144 to 0.087) and the total effect was +0.160 (95% CI, 0.059 to 0.264). The proportion mediated is not reported because the direct effect was negative, which makes that quantity uninterpretable. This analysis rests on the assumption that there is no unmeasured confounding of the mediator–outcome relationship, an assumption that cannot be verified in these data.

Several sensitivity analyses are reported in Supplementary Table S3. Two specifications that retain all 301 patients, using the inverse hyperbolic sine transformation (β = −0.052; *p* = 0.538) and a rank-based normal score (β = −0.134; *p* = 0.284), gave the same conclusions, so the exclusion of the single patient from the primary regression did not affect any result. When POD1 CRP was analysed with the preoperative CRP as a covariate, the robotic approach was associated with a higher POD1 CRP (β = 0.184; *p* < 0.001); after operative time, age and BMI were added, this association was no longer significant (β = −0.051; *p* = 0.365), reproducing the pattern of the primary model. The same pattern was also obtained when the four patients who received neoadjuvant chemotherapy were excluded, when the two patients without demonstrated carcinoma were retained, and when patients with no residual carcinoma in the hysterectomy specimen were additionally excluded.

### 3.5. Subgroup Analysis by BMI

Table 5 presents the CRP rise stratified by BMI. Among normal-weight patients (BMI < 25.0 kg/m^2^; Lap, *n* = 101; RALS, *n* = 33), the CRP rise did not differ significantly between the approaches (2.27 vs. 1.95 mg/dL; *p* = 0.139). A borderline between-group difference was observed in the overweight category (BMI 25.0–29.9 kg/m^2^; Lap, *n* = 58; RALS, *n* = 35), in which RALS was associated with a greater CRP rise (3.36 vs. 2.13 mg/dL; *p* = 0.050). Among obese patients (BMI ≥ 30.0 kg/m^2^; Lap, *n* = 43; RALS, *n* = 31), the CRP rise was numerically higher after RALS but the difference did not reach statistical significance (3.36 vs. 2.70 mg/dL; *p* = 0.226).

The interaction between BMI category and the surgical approach was tested formally and was not statistically significant (F(2, 294) = 0.288; *p* = 0.750); the result was unchanged after adjustment for age and operative time (F(2, 292) = 0.202; *p* = 0.817). The subgroup pattern therefore most probably reflects limited statistical power within strata rather than a genuine BMI-specific effect, and the subgroup analysis is reported as exploratory and hypothesis-generating only. No claim of a BMI-specific effect is made.

## 4. Discussion

We examined patients with endometrial cancer who underwent robot-assisted or laparoscopic minimally invasive hysterectomy. Patients who underwent robot-assisted surgery showed a greater early postoperative rise in CRP. However, after adjustment for operative time the surgical approach was no longer associated with CRP elevation, whereas operative time and BMI were. A formal mediation analysis indicated an indirect effect of the surgical approach on the CRP rise through operative time. These findings are consistent with an early postoperative inflammatory response that tracks the duration and extent of the procedure and patient-related factors rather than a platform-specific effect independent of operative duration, although the design of this study cannot establish that pathway causally.

Studies comparing robot-assisted and laparoscopic surgery for endometrial cancer have primarily examined short-term perioperative outcomes such as estimated blood loss, conversion rate to laparotomy, length of hospital stay, and complication rates. Previous studies have indicated that robotic surgery achieves perioperative outcomes comparable to those of laparoscopic surgery. However, findings regarding operative time have been inconsistent, with institutional characteristics and surgeon proficiency cited as contributing factors [3,5,16,17]. In the present study, operative time, POD1 CRP, and the magnitude of CRP elevation were all higher in the robotic group, and the difference in CRP rise persisted after propensity score matching. Nevertheless, the surgical approach was not independently associated with CRP elevation in adjusted analyses. We emphasise that operative time is not a confounder of the relationship between the surgical approach and the inflammatory response but lies on the causal pathway between them, and that it may itself be determined by the platform, the learning curve, docking and console time, the extent of lymphadenectomy and institutional workflow. The additional time required for docking and console set-up is itself part of what makes robotic procedures longer in our institution. A model containing operative time therefore estimates a direct effect conditional on operative time rather than the total effect of the surgical approach, and attenuation of a regression coefficient is not in itself evidence of explanation. The association between postoperative inflammatory markers such as CRP and IL-6 and the extent of surgical insult is well established [9,10,18], and the present results are consistent with that body of evidence. Evidence from other surgical fields points in the same direction: a randomised comparison of laparoscopic percutaneous internal ring suturing with open repair for paediatric inguinal hernia showed a significantly greater postoperative inflammatory response after open surgery, supporting the view that the extent of tissue manipulation and the duration of the procedure contribute to the magnitude of the postoperative inflammatory response [19].

The RALS group in this cohort comprised a lower-risk population, with a significantly higher proportion of patients with low-risk disease (75.8% vs. 61.3%; *p* = 0.044), and the two groups underwent lymphadenectomy at a comparable rate (54.5% vs. 60.4%; *p* = 0.399) with an identical median number of nodes retrieved. This pattern would be expected to favour the robotic group with respect to surgical burden, which makes case complexity an unlikely principal explanation for the greater CRP elevation observed after RALS, although residual differences in overall case complexity cannot be excluded.

The association between BMI and CRP elevation observed in this study is noteworthy. Robotic surgery increases the success rate of minimally invasive surgery and decreases the need for conversion to laparotomy in obese patients with endometrial cancer [7,20,21,22]. Conversely, obesity may increase the technical complexity of surgical field exposure, port placement, patient positioning, and instrument manipulation, potentially resulting in longer operative times and a greater perioperative burden [23,24]. The present findings may be interpreted as the net result of two opposing components of robotic surgery: improved visualization, tremor filtration, and instrument dexterity may reduce mechanical tissue injury, whereas a prolonged operative time may increase the duration of tissue manipulation, pneumoperitoneum, anesthesia, and physiological stress. Recent randomized and pooled evidence has generally reported lower, rather than higher, postoperative CRP levels after robotic abdominal surgery despite longer operative times. Three concrete differences may account for the contrast with our cohort. Those series were drawn from mature robotic programmes in which operative times were comparable to or shorter than those of laparoscopy, whereas ours includes the earliest phase of implementation; the extent of lymphadenectomy differs between series; and the case mix and body habitus of the populations differ substantially. Whether improving operative efficiency reduces the early postoperative inflammatory response should be evaluated prospectively.

The between-group difference in CRP elevation was of borderline significance only in the overweight category (BMI 25.0–29.9 kg/m^2^). We tested the interaction between BMI category and the surgical approach formally and found it to be non-significant (*p* = 0.750), and we therefore do not interpret the effect as specific to any BMI stratum. The obese subgroup comprised the smallest number of patients (Lap, *n* = 43; RALS, *n* = 31) and was the least powered stratum, no adjustment was made for multiple comparisons, and the subgroup analysis is reported as exploratory and hypothesis-generating only. These findings do not indicate that robotic surgery is disadvantageous for obese patients.

Careful consideration is warranted in the clinical interpretation of CRP levels. Although CRP is a convenient and widely used indicator of the perioperative inflammatory response, it is also commonly used to assess postoperative infectious complications [25,26,27,28]. Because this study evaluated the change in CRP level from the preoperative value to POD1, the findings reflect the degree of the early postoperative inflammatory response rather than serving as a diagnostic indicator of infectious complications. The absolute difference between the groups was modest, approximately 0.6 mg/dL in the median CRP rise, and a difference of this magnitude has no established clinical correlate. We did not demonstrate any association between the CRP difference and postoperative recovery, complications or any patient-centred outcome, and the CRP rise should therefore be regarded here only as a marker of the early inflammatory response and not as a measure of clinical harm. Readmission occurred in three RALS patients and in none of the Lap patients; with only three events, arising from postoperative bleeding, a retroperitoneal abscess and pyelonephritis in patients with relevant comorbidity, these heterogeneous events could not be clearly linked to the early CRP findings of this study, and no inference about the comparative safety of the two approaches should be drawn from them.

This study had several limitations. The most important is that the effect of the robotic platform cannot be separated from the effect of the operating surgeon. Essentially all robotic procedures were performed or supervised by a single attending surgeon, whereas laparoscopic procedures were performed by 13 surgeons, so that surgeon and platform are almost completely collinear and a surgeon-stratified analysis is not possible. The cohort also includes the earliest phase of our robotic programme, during which operative times were longest. Surgeon experience and the learning curve may therefore account for a substantial part of the difference in operative time on which our principal observation rests. Because the robotic platform changed from the da Vinci Si to the da Vinci Xi during the study period and the proportion of robotic cases increased over time, platform, calendar period and accumulating experience are mutually confounded, and our design cannot separate them. Second, the study was retrospective and conducted at a single institution, so unmeasured confounding cannot be excluded; the mediation analysis in particular rests on the assumption of no unmeasured mediator–outcome confounding, which cannot be verified here precisely because surgeon and learning-curve effects are inseparable from operative time in our data. Third, inflammatory assessment was limited to the preoperative and POD1 CRP measurements and did not extend to other inflammatory markers or to the temporal trajectory of CRP. Fourth, the only clinical outcomes available were length of stay and readmission; infectious and other complications were not systematically recorded, so the clinical significance of the observed CRP difference remains unknown. Fifth, the BMI-stratified analyses were exploratory, were based on modest subgroup sample sizes, and were not powered to detect between-group differences within strata. This study nevertheless has strengths: a relatively large consecutive cohort, a propensity score model restricted to preoperative variables with balance reported as standardized mean differences, a formal mediation analysis, and a series of sensitivity analyses showing that the conclusions do not depend on the transformation used or on the definition of the analytic cohort.

## 5. Conclusions

In conclusion, among patients undergoing minimally invasive hysterectomy for endometrial cancer, the greater early postoperative elevation of CRP observed after the robotic approach was associated with a longer operative time and with a higher BMI; the relative contributions of the surgical platform, surgeon experience and the learning curve could not be separated in this cohort. The clinical significance of a difference of this magnitude is unknown, no BMI-specific effect was demonstrated, and these findings should not be taken to indicate a clinical disadvantage of robotic surgery. Because surgeon experience and the learning curve cannot be separated from operative time in this cohort, prospective studies that incorporate serial measurements of inflammatory markers, account for surgeon experience and platform differences, and relate the inflammatory response to patient-centred outcomes are needed to clarify the clinical significance of the associations identified here.

## Figures and Tables

**Table 1:** Patient characteristics by surgical approach.

Variable	Laparoscopy (*n* = 202)	Robotic (*n* = 99)	*p* Value
Age, years	57.0 (51.0–67.0)	54.0 (50.0–62.0)	0.027
Body mass index, kg/m^2^	25.00 (22.37–29.01)	27.19 (21.61–31.05)	0.032
BMI category, kg/m^2^			0.020
<25.0	101 (50.0)	33 (33.3)	
25.0–29.9	58 (28.7)	35 (35.4)	
≥30.0	43 (21.3)	31 (31.3)	
Preoperative CRP, mg/dL	0.10 (0.10–0.20)	0.11 (0.10–0.26)	0.084
Preoperative histology			0.424
Atypical endometrial hyperplasia	6 (3.0)	3 (3.0)	
Endometrioid G1	158 (78.2)	85 (85.9)	
Endometrioid G2	15 (7.4)	6 (6.1)	
Endometrioid G3	5 (2.5)	1 (1.0)	
Serous	15 (7.4)	2 (2.0)	
Carcinosarcoma	1 (0.5)	0 (0.0)	
Not evaluable	2 (1.0)	2 (2.0)	
Preoperative risk category			0.027
Low	159 (79.5)	89 (91.8)	
Intermediate	32 (16.0)	6 (6.2)	
High	9 (4.5)	2 (2.1)	
FIGO stage (2008)			0.472
Stage IA	145 (71.8)	81 (81.8)	
Stage IB	23 (11.4)	5 (5.1)	
Stage II	18 (8.9)	8 (8.1)	
Stage IIIA–IVB	16 (7.9)	5 (5.1)	
Postoperative histology			0.485
Endometrioid G1	153 (75.7)	80 (80.8)	
Endometrioid G2	15 (7.4)	12 (12.1)	
Endometrioid G3	7 (3.5)	2 (2.0)	
Serous	16 (7.9)	4 (4.0)	
Clear cell	1 (0.5)	0 (0.0)	
Carcinosarcoma	3 (1.5)	0 (0.0)	
Atypical endometrial hyperplasia	1 (0.5)	0 (0.0)	
No residual carcinoma	6 (3.0)	1 (1.0)	
Lymphovascular space invasion	25/194 (12.9)	7/99 (7.1)	0.190
Postoperative recurrence-risk category			0.044
Low	122 (61.3)	75 (75.8)	
Intermediate	52 (26.1)	17 (17.2)	
High	25 (12.6)	7 (7.1)	
Lymphadenectomy performed	122 (60.4)	54 (54.5)	0.399
Nodes retrieved, *n* (if performed)	11 (8–16)	11 (7–19)	0.812
Neoadjuvant chemotherapy	4 (2.0)	0 (0.0)	0.307
Robotic platform			
da Vinci Si	—	52 (52.5)	
da Vinci Xi	—	43 (43.4)	
hinotori	—	4 (4.0)	

Continuous variables are presented as medians (interquartile ranges) and were compared using the Mann–Whitney U test. Categorical variables are presented as *n* (%) and were compared using the chi-square test or Fisher’s exact test, as appropriate. The preoperative risk category was adapted from the recurrence-risk classification of the 2023 Japan Society of Gynecologic Oncology guidelines [1] and applied before surgery using the biopsy histology in place of the final histology and the preoperative stage in place of the pathological stage: low, endometrioid G1 or G2 with preoperative stage IA; intermediate, endometrioid G3 or a special histological type with preoperative stage IA, or endometrioid G1 or G2 with preoperative stage IB; high, all other combinations. Atypical endometrial hyperplasia was grouped with endometrioid G1/G2. The recurrence-risk category is assigned from the final surgical pathology and is therefore a postoperative variable. Missing observations were as follows: postoperative recurrence-risk category, 3 (all Lap); lymphovascular space invasion, 8 (all Lap); evaluable preoperative biopsy, 4 (2 in each group). No values were missing for any other variable, and no imputation was performed. Percentages are calculated over patients with non-missing data. A full account of missing data is given in Supplementary Table S1. CRP, C-reactive protein; FIGO, International Federation of Gynecology and Obstetrics.

**Table 2:** Primary and secondary surgical outcomes.

Outcome	Laparoscopy (*n* = 202)	Robotic (*n* = 99)	*p* Value
*Primary outcome*			
CRP rise (POD1 − preoperative CRP), mg/dL	2.13 (1.28–3.50)	2.88 (2.01–4.06)	0.001
*Secondary outcomes*			
Operative time, min	210.5 (176.0–246.0)	271.0 (230.5–347.0)	<0.001
Estimated blood loss, mL	20.0 (10.0–42.8)	23.0 (10.0–52.5)	0.324
Length of hospital stay, days	5.0 (5.0–5.0)	5.0 (5.0–5.0)	0.014
POD1 CRP, mg/dL	2.40 (1.54–3.84)	3.14 (2.15–4.68)	0.001
Readmission, n (%)	0 (0.0)	3 (3.0)	0.035
Preoperative CRP, mg/dL	0.10 (0.10–0.20)	0.11 (0.10–0.26)	0.084

Continuous variables are presented as medians (interquartile ranges) and were compared using the Mann–Whitney U test. Readmission rates were compared using Fisher’s exact test. All three readmissions occurred in the robotic group and were due to postoperative bleeding in a patient receiving corticosteroids, a retroperitoneal haematoma with abscess formation in a patient with poorly controlled diabetes, and pyelonephritis in a further patient with diabetes. The medians for length of hospital stay were identical in the two groups; the statistical difference reflects the distribution within a narrow range and is of no clinical consequence. CRP, C-reactive protein; POD1, postoperative day 1.

**Table 3:** Covariate balance before and after propensity score matching, and outcomes in the matched cohort.

Variable	Laparoscopy	Robotic	SMD
*Before matching (Lap, n = 200; RALS, n = 97)*			
Age, years	58.92 (12.53)	55.73 (9.43)	−0.288
Body mass index, kg/m^2^	26.14 (5.83)	27.83 (6.30)	0.278
Preoperative CRP, mg/dL	0.35 (1.47)	0.30 (0.47)	−0.037
Preoperative risk: low	159 (79.5)	89 (91.8)	0.355
Preoperative risk: intermediate	32 (16.0)	6 (6.2)	−0.316
Preoperative risk: high	9 (4.5)	2 (2.1)	−0.137
Lymphadenectomy	122 (61.0)	54 (55.7)	−0.108
*After matching (Lap, n = 154; RALS, n = 92)*			
Age, years	56.47 (12.43)	55.84 (9.43)	−0.057
Body mass index, kg/m^2^	27.33 (5.80)	27.10 (5.44)	−0.041
Preoperative CRP, mg/dL	0.27 (0.43)	0.29 (0.47)	0.039
Preoperative risk: low	90.2%	91.3%	0.038
Preoperative risk: intermediate	8.2%	6.5%	−0.063
Preoperative risk: high	1.6%	2.2%	0.040
Lymphadenectomy	60.9%	58.7%	−0.044
*Outcomes in the matched cohort*			*p* value
CRP rise, mg/dL	2.22 (1.47–3.52)	2.91 (2.02–4.04)	0.010
Operative time, min	211.5 (176.0–246.0)	284.0 (232.3–348.3)	<0.001
POD1 CRP, mg/dL	2.49 (1.61–3.94)	3.17 (2.16–4.64)	0.008

Propensity scores were estimated by logistic regression using preoperative covariates only: age, body mass index, preoperative CRP, the preoperative risk category, and lymphadenectomy. Matching was performed using a 1:2 nearest-neighbor algorithm without replacement with a caliper of 0.15 × SD of the logit propensity score. Balance is expressed as the standardized mean difference (SMD); an absolute SMD below 0.10 was taken to indicate acceptable balance, and the largest absolute SMD after matching was 0.063. Four patients whose preoperative biopsy was not evaluable could not enter the model, leaving 297 patients eligible for matching. Before matching, continuous covariates are presented as mean (standard deviation) and categorical covariates as *n* (%). Because the number of laparoscopic controls per matched set varies (154 controls for 92 robotic patients), all values after matching, and the standardized mean differences after matching, are weighted by the matching weights; weighted percentages rather than counts are therefore shown for categorical covariates. Outcomes in the matched cohort are presented as medians (interquartile ranges) and were compared using the Mann–Whitney U test without weighting; the weighted matched-set analysis of the primary outcome is reported in the text. Full details of the model are given in Supplementary Table S2 and the Love plot in Supplementary Figure S1. Continuous variables are presented as medians (interquartile ranges) and were compared using the Mann–Whitney U test. CRP, C-reactive protein; SD, standard deviation.

**Table 4:** Multivariable linear regression analysis of log-transformed CRP rise, and mediation analysis with operative time as the mediator.

Variable	β Coefficient	SE	95% CI	*p* Value
Model A: adjusted for operative time (direct effect)				
Robotic (vs. laparoscopic)	−0.0293	0.0597	−0.1463 to 0.0876	0.623
Age (centered)	−0.0006	0.0021	−0.0047 to 0.0034	0.761
Body mass index (centered)	0.0090	0.0042	0.0008 to 0.0173	0.033
Preoperative CRP (centered)	0.0360	0.0594	−0.0805 to 0.1525	0.546
Operative time (centered)	0.0026	0.0004	0.0018 to 0.0035	<0.001
Lymphadenectomy	0.0781	0.0544	−0.0286 to 0.1847	0.152
Intercept	1.2074	0.0477	1.1140 to 1.3008	<0.001
Model B: operative time omitted (total effect)				
Robotic (vs. laparoscopic)	0.1591	0.0537	0.0538 to 0.2643	0.003
Age (centered)	−0.0017	0.0022	−0.0059 to 0.0026	0.447
Body mass index (centered)	0.0147	0.0044	0.0062 to 0.0233	<0.001
Preoperative CRP (centered)	0.0910	0.0621	−0.0308 to 0.2128	0.144
Lymphadenectomy	0.2294	0.0510	0.1294 to 0.3294	<0.001
Intercept	1.0604	0.0433	0.9756 to 1.1451	<0.001
Mediation analysis (mediator: operative time)				
Indirect effect (ACME)	0.192	—	0.120 to 0.278	—
Direct effect (ADE)	−0.032	—	−0.144 to 0.087	—
Total effect	0.160	—	0.059 to 0.264	—

Dependent variable: log (CRP rise + 1); *n* = 300. Continuous covariates were mean-centered. Confidence intervals for the regression models were calculated as β ± 1.96 × SE. Model A includes operative time and therefore estimates a direct effect conditional on operative time; Model B omits operative time and estimates the total effect of the surgical approach. The mediation analysis was adjusted for age, body mass index and lymphadenectomy, with bias-corrected and accelerated bootstrap confidence intervals from 2000 resamples; the proportion mediated is not reported because the direct effect was negative, which makes that quantity uninterpretable. One patient with a negative CRP rise (−16.69 mg/dL, arising from a markedly elevated preoperative CRP of 20.35 mg/dL) was excluded from this model because the logarithmic transformation was undefined. CI, confidence interval; CRP, C-reactive protein; SE, standard error.

**Table 5:** CRP rise stratified by body mass index category.

BMI Category, kg/m^2^	Laparoscopy		Robotic		*p* Value
	*n*	CRP Rise, mg/dL	*n*	CRP Rise, mg/dL	
<25.0 (normal weight)	101	1.95 (0.95–2.93)	33	2.27 (1.90–3.28)	0.139
25.0–29.9 (overweight)	58	2.13 (1.48–4.42)	35	3.36 (2.02–4.39)	0.050
≥30.0 (obese)	43	2.70 (1.94–3.51)	31	3.36 (2.15–4.81)	0.226

CRP rise was defined as the postoperative day 1 CRP level minus the preoperative CRP level. Values are presented as medians (interquartile ranges) and were compared between surgical approaches within each body mass index category using the Mann–Whitney U test. The interaction between BMI category and the surgical approach was tested on log (CRP rise + 1) and was not statistically significant (F(2, 294) = 0.288; *p* = 0.750; after adjustment for age and operative time, F(2, 292) = 0.202; *p* = 0.817). This subgroup analysis is therefore exploratory and hypothesis-generating only. Body mass index categories were based on the World Health Organization (WHO) classification. BMI, body mass index; CRP, C-reactive protein.

## Data Availability

The data presented in this study are available from the corresponding author upon reasonable request. The data are not publicly available because of privacy and ethical restrictions.

## Supplementary Materials

The following supporting information can be downloaded at: https://www.mdpi.com/article/10.3390/jcm15197376/s1, Table S1, missing data, case distribution by calendar year and robotic platform, lymphadenectomy, and cohort derivation; Table S2, propensity score model; Table S3, sensitivity analyses; Figure S1, Love plot of covariate balance before and after propensity score matching.

## Abbreviations

The following abbreviations are used in this manuscript:

BMI	Body mass index
CI	Confidence interval
CRP	C-reactive protein
FIGO	International Federation of Gynecology and Obstetrics
IQR	Interquartile range
Lap	Laparoscopic surgery
MIS	Minimally invasive surgery
POD1	Postoperative day 1
PSM	Propensity score matching
RALS	Robot-assisted laparoscopic surgery
SE	Standard error
WHO	World Health Organization
