# Supplementary material for: Postoperative C-Reactive Protein as a Marker of the Early Inflammatory Response in Robotic Versus Laparoscopic Minimally Invasive Surgery for Endometrial Cancer: A Propensity Score-Matched Analysis
Source: J Clin Med. 2026 Sep 23;15(19):7376. doi: 10.3390/jcm15197376 (PMC13665805; doi:10.3390/jcm15197376)
Supplement: Supplementary file 1 [file jcm-15-07376-s001.zip › Supplementary_Materials_jcm-4538487_2.pdf]

Supplementary Materials

Manuscript jcm-4538487 · Journal of Clinical Medicine

Postoperative C-reactive Protein as a Marker of the Early Inflammatory Response in Robotic versus Laparoscopic Minimally Invasive Surgery for Endometrial Cancer: A Propensity Score-Matched Analysis

Contents: Table S1, missing data, case distribution by calendar year and robotic platform, lymphadenectomy, and cohort derivation; Table S2, propensity score model; Table S3, sensitivity analyses; and the legend for Figure S1, the Love plot of covariate balance before and after propensity score matching. Figure S1 is supplied as a separate file.

Table S1. Missing data, case distribution, and cohort derivation

S1-a. Missing observations

Variable	Laparoscopy (n = 202)	Robotic (n = 99)	Total
Age	0	0	0
Body mass index	0	0	0
Preoperative CRP	0	0	0
POD1 CRP	0	0	0
CRP rise	0	0	0
Operative time	0	0	0
Estimated blood loss	0	0	0
Lymphadenectomy (performed / not performed)	0	0	0
Length of hospital stay	0	0	0
Readmission	0	0	0
FIGO stage	0	0	0
Evaluable preoperative biopsy (preoperative risk category)	2	2	4
Recurrence-risk category	3	0	3
Lymphovascular space invasion	8	0	8

No imputation was performed. Analyses were carried out on complete cases for the variables concerned, and the sample size is stated for every analysis. A blank entry for the lymph-node count in the source database denotes that lymphadenectomy was not performed rather than an unrecorded count; this variable is therefore complete for all 301 patients. "Evaluable preoperative biopsy" denotes a diagnostic endometrial biopsy from which a histological diagnosis could be assigned; the preoperative stage was available for all 301 patients, so the preoperative risk category could not be assigned in these four patients alone, and they could not enter the propensity score model. CRP, C-reactive protein; POD1, postoperative day 1.

S1-b. Cases by calendar year and surgical approach

Year	2018	2019	2020	2021	2022	2023	2024	2025	Total
Laparoscopy	3	22	34	43	26	27	39	8	202
Robotic	6	11	8	7	16	20	24	7	99
Total	9	33	42	50	42	47	63	15	301

Both approaches were performed in every year of the study, although the proportion of robotic cases increased over time. The study period ran from December 2018 to February 2025.

S1-c. Robotic platform by calendar year

Platform	2018	2019	2020	2021	2022	2023	2024	2025	Total
da Vinci Si	6	11	8	7	16	4	0	0	52

Platform	2018	2019	2020	2021	2022	2023	2024	2025	Total
da Vinci Xi	0	0	0	0	0	14	24	5	43
hinotori	0	0	0	0	0	2	0	2	4
Total	6	11	8	7	16	20	24	7	99

The robotic platform changed from the da Vinci Si to the da Vinci Xi during 2023; four hinotori procedures were performed in 2023 and 2025. Because the platform changed part way through the study and this coincided with accumulating surgical experience, platform, calendar period and experience are mutually confounded. A surgeon-stratified analysis was not possible: essentially all robotic procedures were performed or supervised by a single attending surgeon, whereas the laparoscopic procedures were performed by 13 surgeons, so surgeon and platform are almost completely collinear.

S1-d. Lymphadenectomy

	Laparoscopy (n = 202)	Robotic (n = 99)	p value
Lymphadenectomy performed, n (%)	122 (60.4)	54 (54.5)	0.399
Nodes retrieved if performed, median [IQR]	11 [8–16]	11 [7–19]	0.812

Chi-square test with Yates' correction for the proportion (Fisher's exact test, p = 0.384) and Mann–Whitney U test for the number of nodes. The comparable rate of lymphadenectomy and the identical median node count argue against a substantial difference in surgical complexity between the groups. IQR, interquartile range.

S1-e. Derivation of the analytic cohort

Step	Laparoscopy	Robotic	Total
Consecutive minimally invasive hysterectomies, December 2018 – February 2025	206	100	306
Excluded: cervical cancer (n = 2), leiomyosarcoma (n = 1)	203	100	303
Excluded: no carcinoma demonstrated on final pathology (n = 2)	202	99	301
Analytic cohort	202	99	301
Eligible for propensity score matching (evaluable preoperative biopsy)	200	97	297
Matched 1:2, caliper 0.15 SD	154	92	246
Included in the multivariable regression models	—	—	300

Two patients in whom carcinoma was never demonstrated — one with atypical endometrial hyperplasia on both the diagnostic biopsy and the hysterectomy specimen, and one with atypical endometrial hyperplasia on biopsy and no residual lesion in the specimen — were excluded in accordance with the prespecified criterion that a final histological diagnosis other than endometrial cancer was not eligible. These were also the only two patients without an assigned FIGO stage. Patients in whom carcinoma had been demonstrated on the diagnostic biopsy but no residual carcinoma was found in the hysterectomy specimen were retained. One patient with a negative CRP rise (−16.69 mg/dL) was excluded from the regression models only, because the logarithmic transformation was undefined. Sensitivity analyses for each of these decisions are given in Table S3-b. SD, standard deviation.

S1-f. Additional characteristics of the analytic cohort

Characteristic	Laparoscopy (n = 202)	Robotic (n = 99)	Total
Surgery after neoadjuvant chemotherapy, n (%)	4 (2.0)	0 (0)	4
No residual carcinoma in the hysterectomy specimen, n (%)	6 (3.0)	1 (1.0)	7
Preoperative biopsy not evaluable, n (%)	2 (1.0)	2 (2.0)	4

The four patients who underwent surgery after neoadjuvant chemotherapy had stage IIIC2 (n = 1) or stage IVB (n = 3) disease and were all in the laparoscopic group; a sensitivity analysis excluding them is given in Table S3-b.

Table S2. Propensity score model

S2-a. Multivariable logistic regression for the robotic approach

Covariate	Odds ratio	95% CI	p value
Age, per year	0.989	0.966 to 1.012	0.332
Body mass index, per kg/m^2	1.046	1.003 to 1.091	0.037
Preoperative CRP, per mg/dL	0.957	0.692 to 1.323	0.791
Preoperative risk: intermediate (vs. low)	0.360	0.139 to 0.930	0.035
Preoperative risk: high (vs. low)	0.441	0.087 to 2.230	0.322
Lymphadenectomy (yes vs. no)	0.881	0.528 to 1.468	0.626

S2-b. Distribution of the estimated propensity score before matching

	Laparoscopy (n = 200)	Robotic (n = 97)
Median [IQR]	0.320 [0.264–0.363]	0.362 [0.300–0.421]
Range	0.073–0.656	0.115–0.678

S2-c. Caliper selection

Caliper (SD of the logit propensity score)	Matched Laparoscopy	Matched Robotic	Largest absolute SMD after matching
0.20	155	92	0.067
0.15 (adopted)	154	92	0.063
0.10	150	88	0.076

Propensity scores were estimated by multivariable logistic regression with the surgical approach (robotic = 1, laparoscopic = 0) as the dependent variable, using preoperative covariates only. The preoperative risk category was adapted from the recurrence-risk classification of the 2023 Japan Society of Gynecologic Oncology guidelines for the treatment of uterine body neoplasm, which is assigned from the final surgical pathology; the same framework was applied before surgery using the histological diagnosis of the endometrial biopsy in place of the final histology and the preoperative stage in place of the pathological stage: low risk, endometrioid carcinoma G1 or G2 with preoperative stage IA; intermediate risk, endometrioid carcinoma G3 or a special histological type with preoperative stage IA, or endometrioid carcinoma G1 or G2 with preoperative stage IB; and high risk, all other combinations. Atypical endometrial hyperplasia was grouped with endometrioid G1/G2. Lymphadenectomy was retained because it is planned before surgery on the basis of that risk category. The postoperative FIGO stage, which was used in the original submission, was removed because it is not available when the surgical approach is chosen. Matching was 1:2 nearest neighbour without replacement. A caliper of 0.15 standard deviations was adopted because it gave the best balance among those examined. CI, confidence interval; IQR, interquartile range; SD, standard deviation; SMD, standardized mean difference.

Table S3. Sensitivity analyses

S3-a. Alternative outcome specifications

Specification	n	Approach β (p)	Operative time β (p)	BMI β (p)
log (CRP rise + 1) — reference model	300	−0.054 (0.347)	0.00290 (<0.001)	0.0086 (0.036)
Inverse hyperbolic sine of CRP rise	301	−0.052 (0.538)	0.00373 (<0.001)	0.0112 (0.061)
Rank-based normal score of CRP rise	301	−0.134 (0.284)	0.00629 (<0.001)	0.0180 (0.042)
log (POD1 CRP + 1), adjusted for preoperative CRP	301	−0.051 (0.365)	0.00284 (<0.001)	0.0110 (0.006)
log (POD1 CRP + 1), preoperative CRP only	301	0.184 (<0.001)	—	—

A common adjustment set comprising the surgical approach, operative time, age and body mass index was used across all the specifications in this table so that they are directly comparable with one another; the coefficients therefore differ slightly from those of Model A in Table 4, which additionally includes the preoperative CRP and lymphadenectomy. The inverse hyperbolic sine and rank-based normal score transformations are defined for negative values and therefore retain the patient whose CRP rise was −16.69 mg/dL, who had to be excluded from the reference model. When POD1 CRP was adjusted for the preoperative CRP alone, the robotic approach was associated with a higher POD1 CRP; after operative time, age and body mass index were added, the association was no longer significant, reproducing the pattern of the reference model. BMI, body mass index; CRP, C-reactive protein; POD1, postoperative day 1.

S3-b. Alternative definitions of the analytic cohort

Analytic cohort	n	Total effect β (p)	Adjusted β (p)	Interaction p	Matched CRP rise, mg/dL (p)	Largest |SMD|
Primary analysis	301	0.178 (0.002)	−0.054 (0.347)	0.750	2.22 vs. 2.91 (0.010)	0.063
Also excluding neoadjuvant chemotherapy (n = 4)	297	0.184 (0.001)	−0.046 (0.422)	0.647	2.29 vs. 2.91 (0.008)	0.084
Retaining the two patients without demonstrated carcinoma	303	0.172 (0.002)	−0.059 (0.301)	0.732	2.22 vs. 2.88 (0.008)	0.063
Also excluding patients with no residual carcinoma (n = 7)	294	0.186 (0.001)	−0.045 (0.433)	0.778	2.14 vs. 2.74 (0.014)	0.108

The total effect is the coefficient for the surgical approach in a model that omits operative time; the adjusted estimate additionally includes operative time, age and body mass index. The interaction p value is for the interaction between body mass index category and the surgical approach on log (CRP rise + 1). Propensity score matching was repeated within each cohort definition using the same model and a caliper of 0.15 standard deviations, and the matched CRP rise is the median in the laparoscopic and robotic groups respectively. The same pattern — a significant unadjusted difference, a non-significant difference after adjustment for operative time, and no interaction with body mass index — was obtained under every definition, indicating that the eligibility decisions do not influence the conclusions. SMD, standardized mean difference.

Figure legend

Figure S1. Love plot of covariate balance before and after propensity score matching.

Absolute standardized mean differences for every covariate in the propensity score model before (open circles) and after (filled circles) 1:2 nearest-neighbour matching without replacement with a caliper of 0.15 standard deviations of the logit propensity score. The dashed vertical line marks the threshold of 0.10 taken to indicate acceptable balance. All covariates were below the threshold after matching; the largest absolute standardized mean difference was 0.063, for the intermediate preoperative risk category. Before matching, the corresponding values were 0.288 for age, 0.278 for body mass index, 0.037 for preoperative CRP, 0.355, 0.316 and 0.137 for the low, intermediate and high preoperative risk categories, and 0.108 for lymphadenectomy; after matching they were 0.057, 0.041, 0.039, 0.038, 0.063, 0.040 and 0.044 respectively.

The figure itself is supplied as a separate file (FigureS1_jcm-4538487.pptx).
